# A FRET flow cytometry method for monitoring cytosolic and glycosomal glucose in living kinetoplastid parasites

**DOI:** 10.1371/journal.pntd.0006523

**Published:** 2018-05-31

**Authors:** Charles M. Voyton, Yijian Qiu, Meredith T. Morris, P. Christine Ackroyd, Jimmy Suryadi, Logan Crowe, James C. Morris, Kenneth A. Christensen

**Affiliations:** 1 Department of Chemistry, Clemson University, Clemson, South Carolina, United States of America; 2 Department of Chemistry and Biochemistry, Brigham Young University, Provo, Utah, United States of America; 3 Eukaryotic Pathogens Innovation Center, Department of Genetics and Biochemistry, Clemson University, Clemson, South Carolina, United States of America; Hunter College, CUNY, UNITED STATES

## Abstract

The bloodstream lifecycle stage of the kinetoplastid parasite *Trypanosoma brucei* relies solely on glucose metabolism for ATP production, which occurs in peroxisome-like organelles (glycosomes). Many studies have been conducted on glucose uptake and metabolism, but none thus far have been able to monitor changes in cellular and organellar glucose concentration in live parasites. We have developed a non-destructive technique for monitoring changes in cytosolic and glycosomal glucose levels in *T*. *brucei* using a fluorescent protein biosensor (FLII^12^Pglu-700μδ6) in combination with flow cytometry. *T*. *brucei* parasites harboring the biosensor allowed for observation of cytosolic glucose levels. Appending a type 1 peroxisomal targeting sequence caused biosensors to localize to glycosomes, which enabled observation of glycosomal glucose levels. Using this approach, we investigated cytosolic and glycosomal glucose levels in response to changes in external glucose or 2-deoxyglucose concentration. These data show that procyclic form and bloodstream form parasites maintain different glucose concentrations in their cytosol and glycosomes. In procyclic form parasites, the cytosol and glycosomes maintain indistinguishable glucose levels (3.4 ± 0.4mM and 3.4 ± 0.5mM glucose respectively) at a 6.25mM external glucose concentration. In contrast, bloodstream form parasites maintain glycosomal glucose levels that are ~1.8-fold higher than the surrounding cytosol, equating to 1.9 ± 0.6mM in cytosol and 3.5 ± 0.5mM in glycosomes. While the mechanisms of glucose transport operating in the glycosomes of bloodstream form *T*. *brucei* remain unresolved, the methods described here will provide a means to begin to dissect the cellular machinery required for subcellular distribution of this critical hexose.

## Author summary

African sleeping sickness is caused by *Trypanosoma brucei*. Tens of millions of people living in endemic areas are at risk for the disease. Within the mammalian bloodstream, *T*. *brucei* parasites sustain all their energy needs by metabolizing glucose present in the host’s blood within specialized organelles known as glycosomes. *In vitro*, bloodstream parasites rapidly die if glucose is removed from their environment. This reliance on glucose for survival has made glucose metabolism in *T*. *brucei* an important area of study with the aim to develop targeted therapeutics that disrupt glucose metabolism. However, there have previously been no reported methods to study glucose uptake and distribution dynamics in intact glycosomes in live *T*. *brucei*. Here we describe development of approaches for observing changes in glucose concentration in glycosomes in live *T*. *brucei*. Results obtained using these methods provide new insights into how *T*. *brucei* acquires and transports glucose to sustain cell survival.

## Introduction

Glucose is an important metabolite for members of the class Kinetoplastea that includes *Trypanosoma brucei*, *T*. *cruzi*, and parasites of the genus *Leishmania*. Human African trypanosomiasis (HAT) is caused by a bloodstream infection with *T*. *brucei*. The disease is endemic to sub-Saharan Africa where it is estimated that 60 million people live at risk for contracting the disease [1]. Despite the millions of people at risk, adequate treatment options, especially for late stage disease, are lacking, and can be accompanied by severe drug toxicity [2]. In addition, resistance to existing drugs has been reported [3], [4]. For these reasons, we are expanding the repertoire of analytical methods used to study *T*. *brucei* metabolism to advance our understanding of HAT and enable development of new and more effective anti-trypanosomal treatment(s).

The parasitic Kinetoplastea differ from one another in several key ways. First, *T*. *brucei* and *Leishmania* parasites spread by biting flies (the tsetse fly and sandfly, respectively) during bloodmeals, while *T*. *cruzi* is deposited in the feces of blood feeding Triatominae bugs. While *T*. *brucei* is exclusively an extracellular parasite, *T*. *cruzi* and *Leishmania spp*. inhabit intracellular niches in the amastigote life cycle stage. Nevertheless, the mechanisms of acquisition, subcellular distribution and metabolism of glucose are largely conserved in the kinetoplastid parasites when compared to the complementing machinery of their mammalian hosts, suggesting commonalities that may yield potential therapeutic targets that are pan-kinetoplastid.

The bloodstream form (BSF) of *T*. *brucei* relies on glucose metabolism in the mammalian host’s blood for ATP production and does not use other means of ATP production such as mitochondrial respiration or the Krebs cycle. When denied glucose, BSF parasites die rapidly [5]. The procyclic or insect stage (PCF) parasite harbors a more complete mitochondrial protein repertoire and is capable of metabolizing other carbon sources like amino acids and fatty acids, in addition to glucose [6]. However, PCF parasites will catabolize glucose preferentially over amino acids and other carbon sources, even if the glucose is present in much lower concentrations [7]. The parasite's reliance on glucose for survival make glycolysis, glucose transport, and the enzymes involved therein an important topic of study.

Importantly, kinetoplastid parasites house the majority of glycolytic enzymes in a unique kinetoplastid-specific organelle, the glycosome. Glycosomes are related to mammalian peroxisomes based on conserved biosynthetic mechanisms and general morphology [8],[9]. While related, the functions of these organelles are not entirely overlapping; the glycosome is unusual in that is harbors the first seven glycolytic enzymes. As a result, glucose metabolism requires glucose transport from the external environment through the cell membrane into the cytosol, and subsequently through the glycosomal membrane into the glycosome before glucose metabolism can occur. The glycosomal localization of glucose metabolism means that glucose transport into the glycosome is inextricably linked to glucose metabolism in both BSF and PCF parasites. Plasma membrane glucose transporter proteins known as trypanosome hexose transporters (THTs) are responsible for movement of glucose across the plasma membrane into the cytosol. However, these molecules have not been detected in glycosomal membranes [10], and the existence and identity of glucose transporter proteins responsible for movement of the hexose into glycosomes remain unresolved [11],[8].

With the advent of green fluorescent protein (GFP) variants in the 1990s, Förster Resonance Energy Transfer (FRET) technologies using these proteins have been useful [12] in the development of recombinant protein biosensors that can detect biologically relevant analytes, including glucose, ATP, calcium and pH [13][14]. These biosensors commonly consist of a FRET pair flanking a protein binding domain specific for a particular analyte. In the case of the glucose FRET sensor FLII^12^Pglu-700μδ6, used here, an ECFP/mCitrine FRET pair flanks an *E*. *coli* periplasmic glucose binding protein [15]. Upon glucose binding, this domain undergoes a conformational change that alters the spatial relationship and orientation of the fluorescent proteins to cause a measurable change in energy transfer between the two fluorophores. This change can be observed in living cells proportional to analyte concentration, allowing glucose to be monitored in real time.

In this study, we have used ratiometric fluorescent protein biosensors to monitor glucose concentration in the cytosol and glycosomes of PCF and BSF *T*. *brucei* and to measure glycosomal glucose changes in response to changes in environmental glucose levels. Results from this work suggest BSF *T*. *brucei* glycosomes maintain glucose levels higher than those present in the cytosol. This observation, and the lack of recognizable glycosomal glucose transporters, suggests that the glucose uptake and distribution machinery may be kinetoplastid-specific and therefore potential therapeutic targets.

## Methods

### Chemicals and reagents

2-deoxyglucose (2-DOG), β-escin, Triton X-100, digitonin and all buffer and cell culture media components were purchased from Sigma (St. Louis, MO). The Q5 mutagenesis kit, all restriction enzymes, and other enzymes and reagents used for cloning were purchased from New England Biolabs (Ipswich, MA). Human T Cell nucleofector kits used for BSF expression vector transfection were purchased from Lonza (Basel, Switzerland). IPTG, carbenicillin and all other drugs used for selection were purchased from Gold Bio (Olivette, MO). To normalize experiments between PCF and BSF cells and to mitigate media effects on cells, studies were carried out in phosphate buffered saline (PBS; 137mM NaCl, 2.7mM KCl, 10mM Na_2_HPO_4_, and 1.8 mM KH_2_PO_4_) adjusted to pH 7.4, and containing glucose at the concentration required for the experiment. pH calibration buffer used for intracellular FLII^12^Pglu-700μδ6 calibration was carried out in HEPES and MOPS buffered saline (HM-PBS; 137mM NaCl, 2.7mM KCl, 25mM MOPS, 25mM HEPES, 10mM Na_2_HPO_4_, and 1.8 mM KH_2_PO_4_) adjusted to the desired pH, and titrated with glucose to the desired concentration for calibration.

### Trypanosome culture

BSF parasites were cultured in HMI-9 media at 37°C in 5% CO_2_ and maintained fastidiously at cell densities between 5x10^4^ and 5x10^6^ cell/mL [16]. PCF form parasites were maintained at 29°C in 5% CO_2_ in SDM-79 media and maintained at cell densities between 5x10^5^ and 1x10^7^ [17].

### Sensor cloning and bacterial expression

The fluorescent biosensor pRSET-FLII^12^Pglu-700μδ6 [15] was acquired from Addgene (Addgene plasmid # 13568). For bacterial expression, chemically competent NEB T7 Express (BL21 strain) cells were transformed with pRSEt-FLII^12^Pglu-700μδ6. Protein was expressed using methods adapted from [18]; briefly, cells were grown in LB broth at 30°C to density of OD_600_ ~0.6 and then induced with 0.7mM IPTG and grown at 18°C for 24 hours. Cells were then harvested and lysed via French press and protein purified using Ni-NTA chromatography (GE; Pittsburg, PA). Purified protein was exchanged into PBS with 20%v/v glycerol using a PD-10 desalting column (GE; Pittsburg, PA) and then stored at -80°C until further use. FLII^12^Pglu-700μδ6 was cloned into the trypanosome expression vectors pXS2 and pXS6 using BamHI and HindIII restriction sites.

### Trypanosome transfection

Using the expression vectors pXS2 or pXS6 respectively, PCF and BSF parasites were transfected with an endogenously expressed fluorescent glucose sensor FLII^12^Pglu-700μδ6 to monitor cytosolic glucose concentration. FLII^12^Pglu-700μδ6 with a type one peroxisomal targeting sequence (PTS-1) added to the C-terminus (GGAKL) via site directed mutagenesis was used to monitor glycosomal glucose levels herein referred as FLII^12^Pglu-700μδ6-PTS (Forward: cgcgaaactgTAAAAGCTTGATCCGGCTG, Reverse: ccgcctttcgcCTTGTACAGCTCGTCCATG). Constructs were transfected into parasites as described [19],[20].

### Microscopy

All live cell DIC and fluorescence microscopy was carried out on an inverted epifluorescence microscope (Olympus IX73; Tokyo, Japan). FRET images were taken using a high speed DG-4 light source (Sutter; Novato, CA) and a sensitive CMOS camera (Hamamatsu Orca-Flash 4; Japan) following excitation 433/30 nm for ECFP and mCitrine or ECFP emission collected using 530/30 and 480/30 nm filters, respectively. All microscope components were controlled and images collected and analyzed using Slidebook 6.0 (Intelligent Imaging Innovation; Denver, CO). To minimize background fluorescence caused by media components, cells were imaged in PBS supplemented with 10 mM glucose (images obtained within 1 hour). Fluorescent intensities were calculated by subtracting the background fluorescence from a cell free field for each image field taken.

Immunofluorescence (IF) slides were prepared using a protocol modified from a previous report [21]. In short, trypanosomes in log phase were harvested by centrifugation (2000 rpm, 10 min), washed with PBS, and resuspended in 1:1 PBS and fix solution (4% formaldehyde in PBS, 100 μL per sample). Cells were loaded to slides and allowed to settle for 30 minutes. Cells were then washed with wash solution (0.1% normal goat serum in PBS), permeabilized with 0.5% Triton in PBS for 30 minutes, and blocked with blocking buffer (10% normal goat serum and 0.1% Triton in PBS) for 30 minutes. Primary and secondary antibodies in blocking buffer were applied to the cells for 1 hour respectively with washes in between. Anti-Aldolase (1:2,000) and anti-GFP (M3E6) (1:200) antisera were used to detect glycosome and PT-FLII^12^Pglu-700μδ6 respectively. Primary antibodies were detected with Alexa Fluor 488-conjugated anti-rabbit (1:1,000) or Alexa Fluor 568-conjugate anti-mouse (1:1,000) antisera. The nucleus and kDNA were stained using VECTASHIELD Antifade Mounting Medium with DAPI (Vector Laboratories; Burlingame, CA). IF slides were visualized using Zeiss Axiovert 200M microscope.

### Protease protection assay

PCF cells (1 x 10^6^) expressing the FLII12Pglu-700μδ6-PTS glucose sensor were harvested by centrifugation (800g, 10 minutes), washed with PBS, and then washed again with STE buffer (250mM sucrose, 25mM Tris-HCl, pH7.4, 1mM EDTA). Pellets were resuspended in STEN buffer (STE with 150mM NaCl) and 0.1mM PMSF and cells permeabilized with digitonin (0.02mg/mL final concentration) by incubation at room temperature (4 minutes). The sample was then centrifuged (20,000 x g, 2 minutes) and resuspended in 85ul STEN buffer with either water or Triton-X100 (1% v/v final concentration) added before addition of water or proteinase K (0.1 ug/uL final concentration). Samples were incubated on ice (30 minutes) and the reaction halted by addition of TCA (10% w/v) followed by centrifugation (20,000 x g, 2 minutes). The pellet was then washed with cold acetone and solubilized in SDS PAGE running buffer. Following resolution by SDS-PAGE and transfer to nitrocellulose, FLII12Pglu-700μδ6-PTS fragments were identified using anti-eYFP antisera.

### *In vitro* characterization of biosensors

Emission spectra of purified FLII^12^Pglu-700μδ6 and FLII^12^Pglu-700μδ6-PTS and glucose titration curves were obtained using a fluorometer (PTI; Edison, NJ) with ECFP excitation at 433 (5-nm bandpass) and emission (5-nm bandpass) of ECFP (450-470nm) and mCitrine (520-540nm) collected. All measurements were performed in PBS and glucose calibration curves obtained for 1–50,000 μM glucose. The FRET ratio was calculated from the sensitized emission of mCitrine (520-540nm) and donor emission of ECFP (450-470nm) emission and plots of glucose FRET ratios relative to glucose concentration were generated using Sigmaplot with values for sensors determined using nonlinear regression for single-site ligand binding. To explore the specificity of FLII^12^Pglu-700μδ6 for glucose and assess its sensitivity toward glucose-6-phosphate, the product of hexokinase which is the first glucose modifying enzyme in glycolysis, purified sensor was incubated with varying concentrations of glucose or glucose-6-phosphate and the resulting change in FRET ratio was obtained using a fluorometer as described above.

### FRET flow cytometry

All flow cytometry experiments were performed on a BD Attune flow cytometer (BD; Walthman, MA) equipped with violet (405nm) and blue (488nm) excitation lasers. mCitrine (530/30) and sensitized emission FRET (530/30) emission were excited with a 488nm and 405nm laser respectively. The FRET ratio was calculated on a cell by cell basis by programming a custom parameter (FRET/mCitrine ratio) using the BD attune cytometry software. Data was analyzed using FlowJo (FlowJo LLC, Ashland, OR) flow cytometry analysis software. To ensure accurate FRET measurements, dead cells and doublets were excluded from analysis using the forward and side scattering characteristics of the cells. With dead cells and doublets removed, cells were then gated based on their fluorescent intensity and only cells that were significantly brighter then non-transfected controls were used for analysis.

### PCF trypanosome selective permeabilization to glucose and *In vivo* FLII^12^Pglu-700μδ6 glucose calibration

*In vivo* glucose calibration of FLII^12^Pglu-700μδ6 was performed on permeabilized cells incubated with varying glucose concentrations to ensure that the whole dynamic range of the sensor was assessed (0.025–50 mM glucose). In these experiments, PCF cells were harvested, washed three times in PBS, and then incubated with glucose in PBS in the presence of 45 μM β-escin for 30 minutes followed by FRET analysis. Permeabilized cells were identified in the analysis a being positive for both propidium iodide and mCitrine. Dissociation constants were obtained from non-linear regression as described above. Glucose dose response curves were generated at different pH values by incubating cells in HM-PBS with 45μM β-escin with varying glucose.

### Hexokinase inhibition assay

In a 96-well microplate, recombinant trypanosome hexokinase 1 (TbHK1) and recombinant glucose sensor were mixed in a buffer containing 50 mM TEA-HCl pH 7.4, 33 mM MgCl_2_, and 10 mM Glucose and incubated for 15 minutes at room temperature. The TbHK1 reaction was then initiated by addition of reaction buffer (50 mM TEA-HCl pH 7.4, 33 mM MgCl_2_, 2U G6PDH (Yeast, Alfa Aesar), 4 mM ATP, 1.5 mM NADP^+^. The activity of TbHK1 was determined from the change in absorbance at 340 nm using Synergy H1 Hybrid Multi-Mode Reader (Biotek).

### 2-DOG inhibition of glucose uptake

To determine the effect of glucose uptake inhibitors on intracellular glucose, parasites expressing biosensors were exposed to buffers containing varying 2-DOG concentrations. In short, parasites were harvested during mid log phase by centrifugation at 1250 x g for 5 minutes and washed 3 times with PBS. Cells were then incubated in PBS supplemented with 10 mM glucose with the addition of 2-DOG concentrations between 0.05–50 mM. Parasites were incubated with 2-DOG (29°C 5% CO_2_ for PCF, 37°C 5% CO_2_ for BSF) for 30 minutes to allow intracellular glucose concentration to stabilize. Parasites were then analyzed via flow cytometry as described herein to determine the resultant FRET/Citrine ratio. FRET/mCitrine ratios were then plotted and fitted as described above.

### Glucose deprivation response

Comparison of glucose uptake under varying environmental glucose concentrations was carried out by incubating cells in glucose concentrations that would allow for the full dynamic range of the biosensor to be interrogated (0.025 and 50mM glucose). Briefly, parasites were harvested while in mid log phase growth and then washed 3 times with PBS to remove media components and normalize glucose concentrations. Washing steps were conducted as quickly as possible (≈2 minutes per wash) to attempt to maintain the cell viability of BSF parasites. Washed cells were exchanged into buffer containing glucose at a desired concentration (0.025 and 50mM), followed by a 30-minute incubation to allow intracellular glucose levels to reach a steady state. The resultant FRET/mCitrine ratios were then collected using flow cytometry and correlated to external glucose concentration. Dose response curves were then fitted assuming a variable Hill slope to yield an apparent K_d_ for glucose binding to sensor expressed in the cytosol.

The apparent in vivo K_d_ for sensor glucose binding determined above was determined under conditions of cell permeability and was used as the basis for relating the sensor response to intracellular glucose concentration. Using the calculated apparent K_d_, intracellular glucose concentrations were calculated in response to external glucose concentration using Eq 1, where *K*_*d*_ is the calculated intracellular K_d_ at pH 7, *R* is the FRET/mCitrine ratio of the experimental point, and *RH* and *RL* were the highest and lowest FRET/mCitrine observed intracellular ratio under high and low glucose conditions.

$$[Glucose]=Kd\frac{\left(R-RL\right)}{\left(RH-R\right)}$$ (1)

Values within one order of magnitude of the intracelluar K_d_ were considered in the linear range of the biosensor; hence, points below 0.195mM and points above 6.25mM extracellular glucose were not considered for calculation. Significance between cytosolic and glycosomal glucose concentration were calculated using one-way ANOVA analysis, with significance threshold p <0.05 for n = 3 replicates.

## Results

### Biosensor expression, localization and characterization in *T*. *brucei*

**Fig 1:**
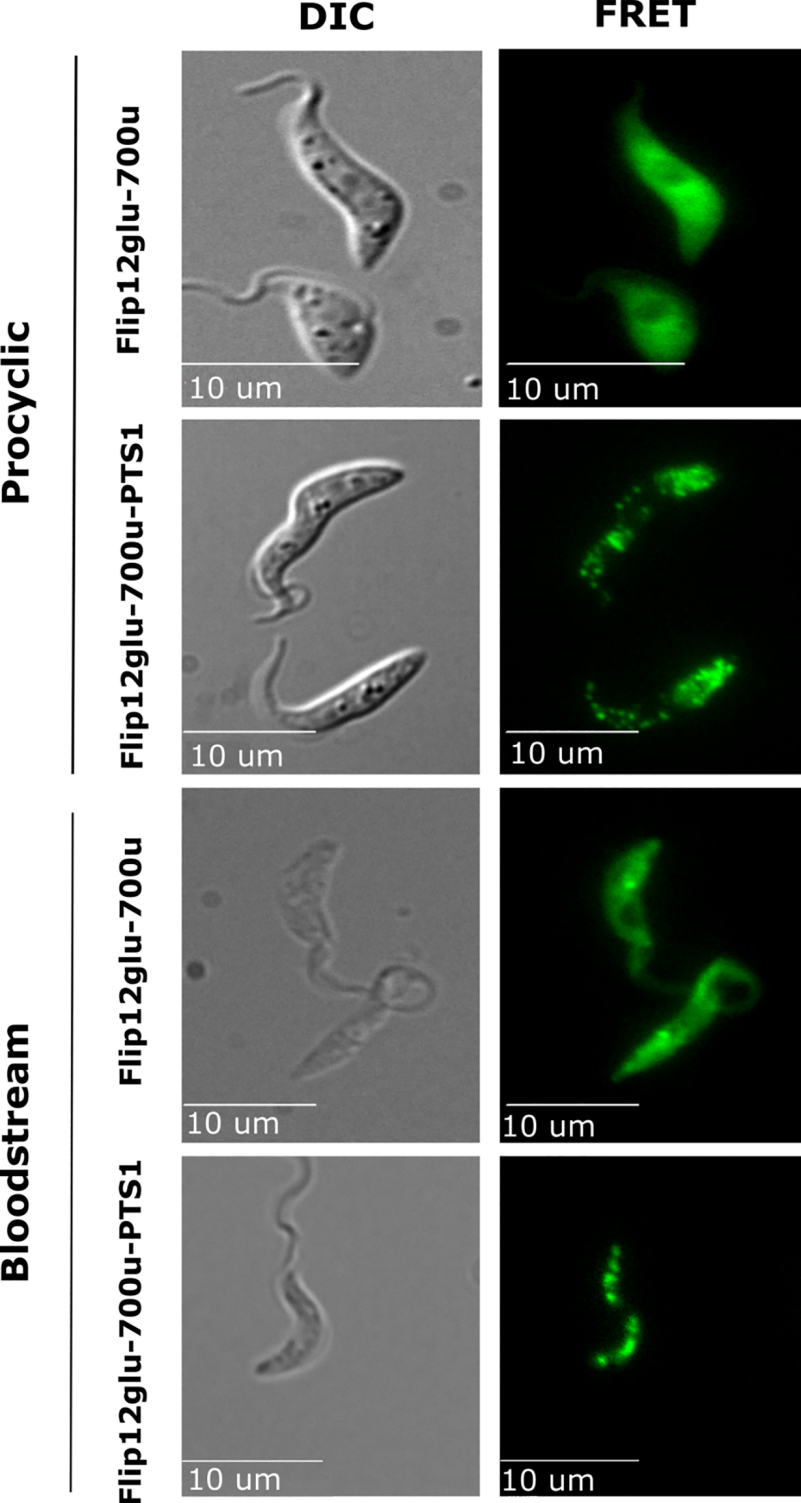
*In vivo* Expression of FLII^12^Pglu-700μδ6 and FLII^12^Pglu-700μδ6-PTS in PCF and BSF T. brucei. DIC (LEFT), and FRET emission (440/20nm ex. 530/30nm em.) (RIGHT) are shown for BSF and PCF *T*. *brucei* cells expressing FLII^12^Pglu-700μδ6 cytosolically, and FLII^12^Pglu-700μδ6-PTS localized in glycosomes. These expression and localization profiles allow us to monitor the glucose changes in labeled glycosomes which only represents the population of glycosomes capable of importing PTS tagged cargos. Cells shown were grown under standard growth conditions in normal SDM-79 or HMI-9 media for PCF and BSF respectively, and exchanged into PBS with 5mM glucose for imaging. Fluorescent images were background subtracted from a cell-free region for every image acquisition.

To monitor glucose dynamics in the cytosol of living trypanosomes, a trypanosome expression vector (either pXS2 or pXS6, for PCF and BSF, respectively [22]) bearing the fluorescent protein biosensor FLII^12^Pglu-700μδ6 was transfected into parasites and stable transformants were selected. Fig 1 demonstrates that this sensor is expressed throughout the cytosol. In kinetoplastids, including *Trypanosoma spp*. and *Leishmania spp*., proteins tagged with a peroxisomal targeting sequence (PTS) are trafficked to the glycosome [23]. To deliver the biosensor to the glycosome, we appended a C-terminal PTS-1 signal sequence to the FLII^12^Pglu-700μδ6 (designated as FLII^12^Pglu-700μδ6-PTS). As shown in Fig 1, FLII^12^Pglu-700μδ6-PTS localizes to small vesicular organelles, consistent with glycosome morphology. To confirm glycosomal localization of FLII^12^Pglu-700μδ6-PTS, parasites were fixed and stained with antisera to the glycosome-resident protein aldolase [24]. Data shown in S1 Fig suggest that FLII^12^Pglu-700μδ6-PTS is localized in glycosomes.

An additional control experiment was performed to ensure that the glucose sensor was delivered to the glycosomal lumen and not simply associating with glycosomal import machinery. A protease protection assay was performed (S2 Fig). Proteolytic fragments of the sensor were only found in the intraglycosomal fraction with none being found associated with the external glycosomal membrane. These results are in agreement with literature showing that large fully folded proteins and protein complexes are imported into the organelle[25].

### PTS-1 biosensor *in vitro* characterization

To ensure that appending a PTS-1 to the biosensor did not impact glucose binding, we measured the affinity of bacterially-expressed and purified FLII^12^Pglu-700μδ6-PTS for glucose via the FRET response of the sensor *in vitro*. Changes in biosensor response were recorded as a function of varied glucose concentrations (S3 Fig). The observed K_d_ value (880 ± 50μM) was in good agreement with previously published values [15], verifying that PTS addition to the C-terminus does not affect sensor response.

FLII^12^Pglu-700μδ6 has previously been shown to not interact with other hexose sugars but binding to glucose-6-phospate, following the first enzymatic step of glycolysis, has not been reported. S4 Fig shows that the FRET/mCitrine ratio observed when FLII^12^Pglu-700μδ6 was incubated with glucose-6-phosphate is not dose dependent. The lack of response to glucose-6-phosphate, which is likely a consequence of the large size and high charge density of the 6-substituted phosphate moiety, ensures that changes in sensor response only result from glucose binding.

### Biosensor characterization in *T*. *brucei*

Flow cytometry is ideal for making FRET measurements because dead and dividing cells can easily be eliminated from analysis. S5 Fig outlines the gating scheme used to identify cells suitable for analysis with dividing doublets being removed by forward scatter characteristics and dead cells by forward and side scatter signal. Live/dead discrimination using forward and side scatter was confirmed using propidium iodide (PI) staining; PI was not used in further experiments to reduce interference with FRET biosensor emission.

**Fig 2:**
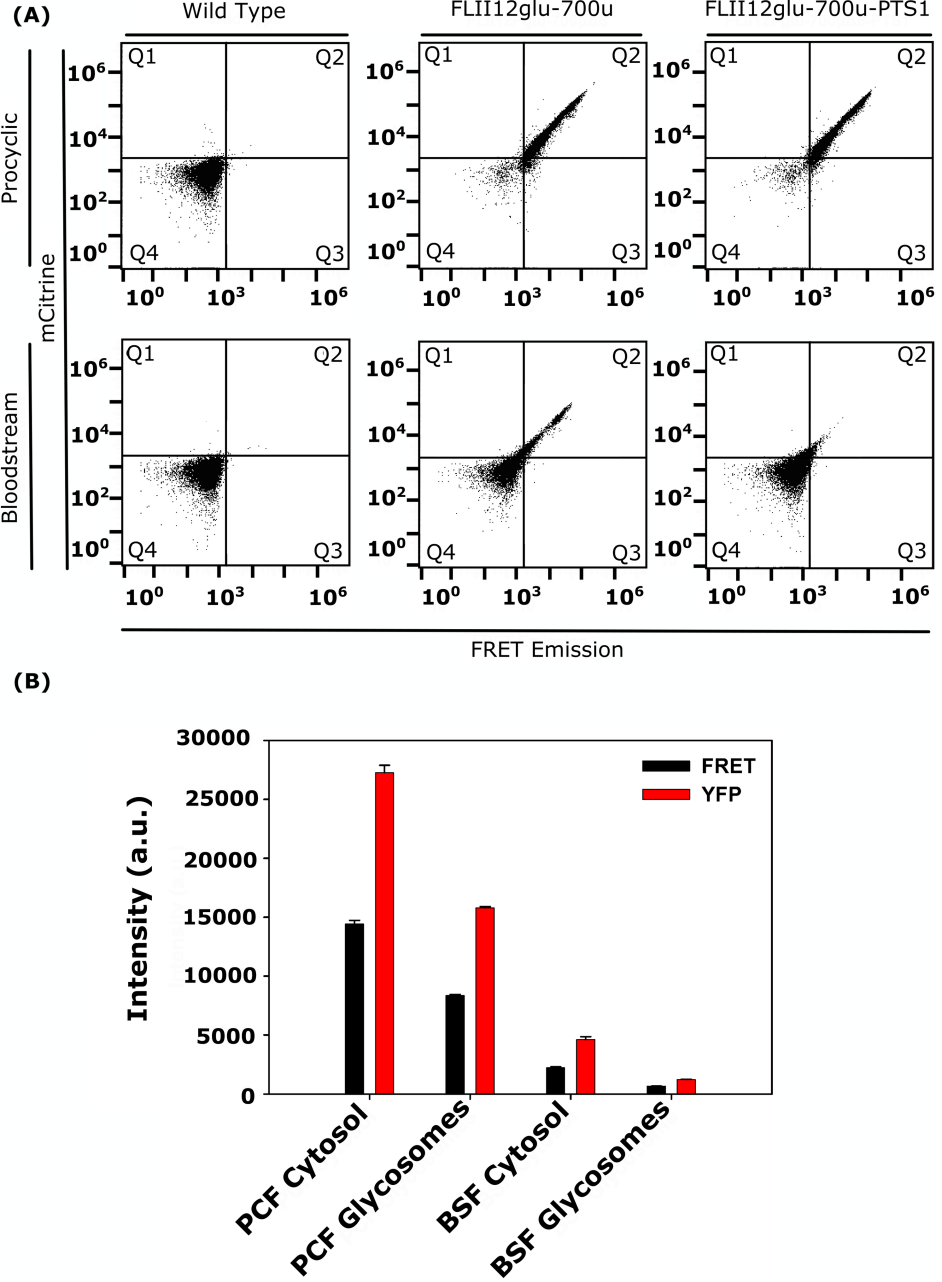
Spectral properties of FLII^12^Pglu-700μδ6 and FLII^12^Pglu-700μδ6-PTS expressed in BSF and PCF parasites can be measured with flow cytometry. (A) Bivariate flow cytometry dot plots representing BSF and PCF *T*. *brucei* expressing FLII^12^Pglu-700μδ6 and FLII^12^Pglu-700μδ6-PTS. X-axis represents FRET emission (405nm excitation 530/30 emission); Y-axis represents mCitrine emission (488nm excitation 530/30nm emission). Wild Type cells not expressing biosensor are used for placing the quadrant gate (Left column). Cells in quadrant 2 (Top right quadrant) are expressing high levels of FLII^12^Pglu-700μδ6 (Middle Column) or FLII^12^Pglu-700μδ6-PTS (Right Column) and are used for FRET analysis. Dead cells, doublets, and the population of cells not robustly expressing biosensor were excluded from FRET analysis. (B) The emission intensities of FRET emission (405nm ex 530/300nm em) and mCitrine (488nm ex 530/30nm em) of FLII^12^Pglu-700μδ6 and FLII^12^Pglu-700μδ6-PTS expressed in PCF and BSF *T*. *brucei* cytosol and glycosomes. Fluorescent emission values were collected via flow cytometry for cells incubated in PBS supplemented with 5mM glucose. Black bars represent FRET emission and red bars represent mCitrine emission, error bars represent the standard deviation of n = 5 replicates.

Flow cytometry was used to measure the mCitrine and FRET fluorescence of parasites expressing FLII^12^Pglu-700μδ6 or FLII^12^Pglu-700μδ6-PTS. To make FRET biosensor measurements it is necessary to excite the donor (ECFP) and acceptor (mCitrine) independently while monitoring the resultant fluorescence of the acceptor at each excitation wavelength [26]. Using the 405nm and 488nm laser of a flow cytometer to excite ECFP and mCitrine respectively, we were able to monitor the FRET and mCitrine fluorescence intensity of parasites expressing FLII^12^Pglu-700μδ6 in cytosol and glycosomes. When analyzed by flow cytometry, PCF and BSF parasites expressing the sensors yielded fluorescent profiles distinct from the parental (untransformed) line (Fig 2A). The observed emission intensity of mCitrine was roughly 2 times higher than the sensitized FRET emission in cells incubated in 10 mM glucose (Fig 2B). This result was in part anticipated because FRET is less efficient than direct excitation due to the inefficiency of dipole-dipole energy transfer of fluorescent protein fusions. It should be noted that while the FRET intensity from BSF parasites was ~10 times lower than that from PCF cells, BSF parasites were still sufficiently bright to allow measurement of changes in FRET ratio.

Intracellular calibration of FRET sensors is preferred, when possible, to account for potential changes in sensor response within the intracellular environment, as well as to determine if autofluorescence from cellular components impacts observed FRET ratios. Though intracellular calibration is common practice when using calcium and pH biosensors, the challenge of calibration when glucose FRET sensors are used for intracellular measurements has limited its practice [27],[28]. This is primarily because, unlike calcium and pH, there are no known compounds that allow glucose to equilibrate across the plasma membrane. Most commonly, *in vitro* calibration curves are used to interpolate the FRET ratio obtained from a sensor expressed in cells [13].

**Fig 3:**
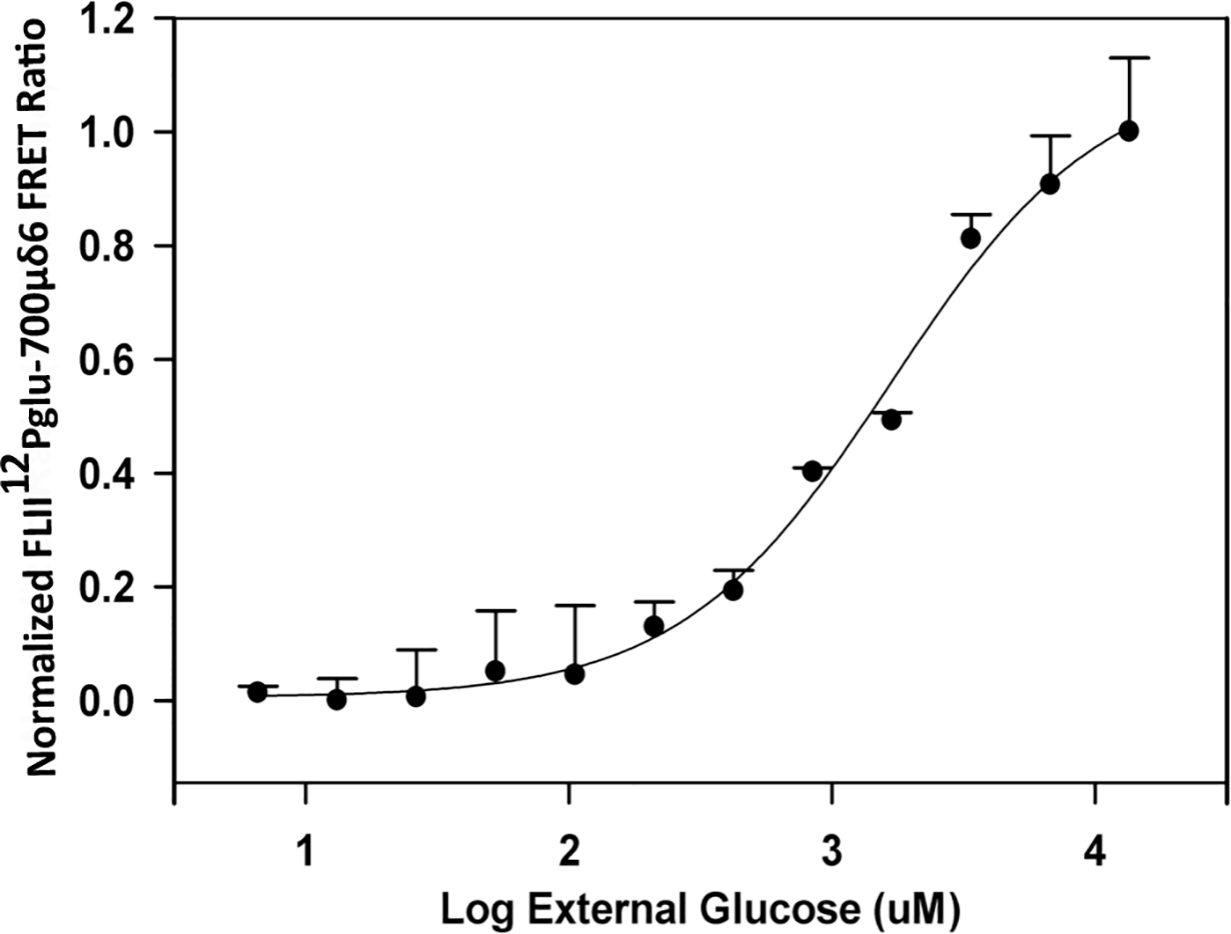
*In vivo* FLII^12^Pglu-700μδ6 glucose calibration curves. Glucose dose response of PCF parasites expressing FLII^12^Pglu-700μδ6. PCF cells expressing FLII^12^Pglu-700μδ6 were permeabilized to glucose using 45μM β-escin and titrated with different glucose concentrations. After intracellular glucose equilibrates with external concentration (30 minutes), the resultant FRET/mCitrine ratio was measured using flow cytometry. Sigmoid curves were fitted using single site ligand binding equations, K_d_ values were calculated as 1000 ± 90 μM. Error bars represent the standard deviation for n = 3 replicates.

To measure the K_d_ of the biosensor in the cytosol, parasites were first incubated with varying concentrations of β-escin, a mild detergent that gently permeabilizes cell membranes [29] and then stained with propidium iodide (PI) to assess cell permeability to small molecules. A treatment with 45 μM β-escin allowed small molecules (glucose and PI) to enter the cell freely while larger molecules (FLII^12^Pglu-700μδ6, other proteins) remained inside the cell (S6 Fig). Using these optimized permeabilization conditions, we carried out an intracellular glucose calibration that yielded a sensor K_d_ = 1000 ± 90 μM (Fig 3). This K_d_ value, which differs only slightly from previously reported values generated *in vitro* [15] (including our own), indicated that the intracellular environment had little impact on FLII^12^Pglu-700μδ6 binding affinity, consistent with the behavior of other intracellular fluorescent protein sensors [13]. Ideally, a glycosomal calibration would have been conducted as well, but methods used for glycosomal permeabilization (i.e. digitonin or β-escin) quench the fluorescence of the sensor at needed concentrations. As a compromise we used the intracellular K_d_ was used as the basis for comparison for all subsequent intracellular and glycosomal experiments. In addition, inclusion of the glucose sensor in an *in vitro* assay of recombinant trypanosome hexokinase (TbHK1) did not significantly alter enzyme activity even when the sensor was present in 10-fold excess over hexokinase (S7 Fig). This suggests that expression of the sensor in the glycosome would not interfere with the activity of the glycolytic enzymes therein [30].

PCF parasite glycosomes acidify in response to nutrient deprivation [32]. Additionally, fluorescent proteins can have pH dependent shifts in their excitation and emission spectra [33], which could affect glycosome measurements. To mitigate pH sensitivity, the glucose sensor FLII^12^Pglu-700μδ6 was generated with the pH- and chloride-insensitive yellow fluorescent protein mCitrine [33], [15]. To further demonstrate that changes in pH do not cause an artificial response in the glucose sensor, we measured the sensitivity of the intracellular glucose sensor to pH, over the range of pH that could be induced by glucose deprivation [32]. Cells expressing FLII^12^Pglu-700μδ6 were permeabilized and an intracellular calibration for glucose was assessed at relevant pH concentrations (Table 1). No pH sensitivity was observed, suggesting that mild acidification of glycosomes during glucose starvation does not impact observed FRET ratio.

**Table 1:** Binding constants for FLII^12^Pglu-700μδ6 biosensor in different environments.

	Calculated K_d_ (μM)
Sensor Calibration	
FLII^12^Pglu-700μδ6 *in vitro;* pH 7	880 ± 50
FLII^12^Pglu-700μδ6 *intracellular;* pH 7	1000 ± 90
FLII^12^Pglu-700μδ6 *intracellular;* pH 6	1000 ± 110

### FRET biosensor response to external environment

**Fig 4:**
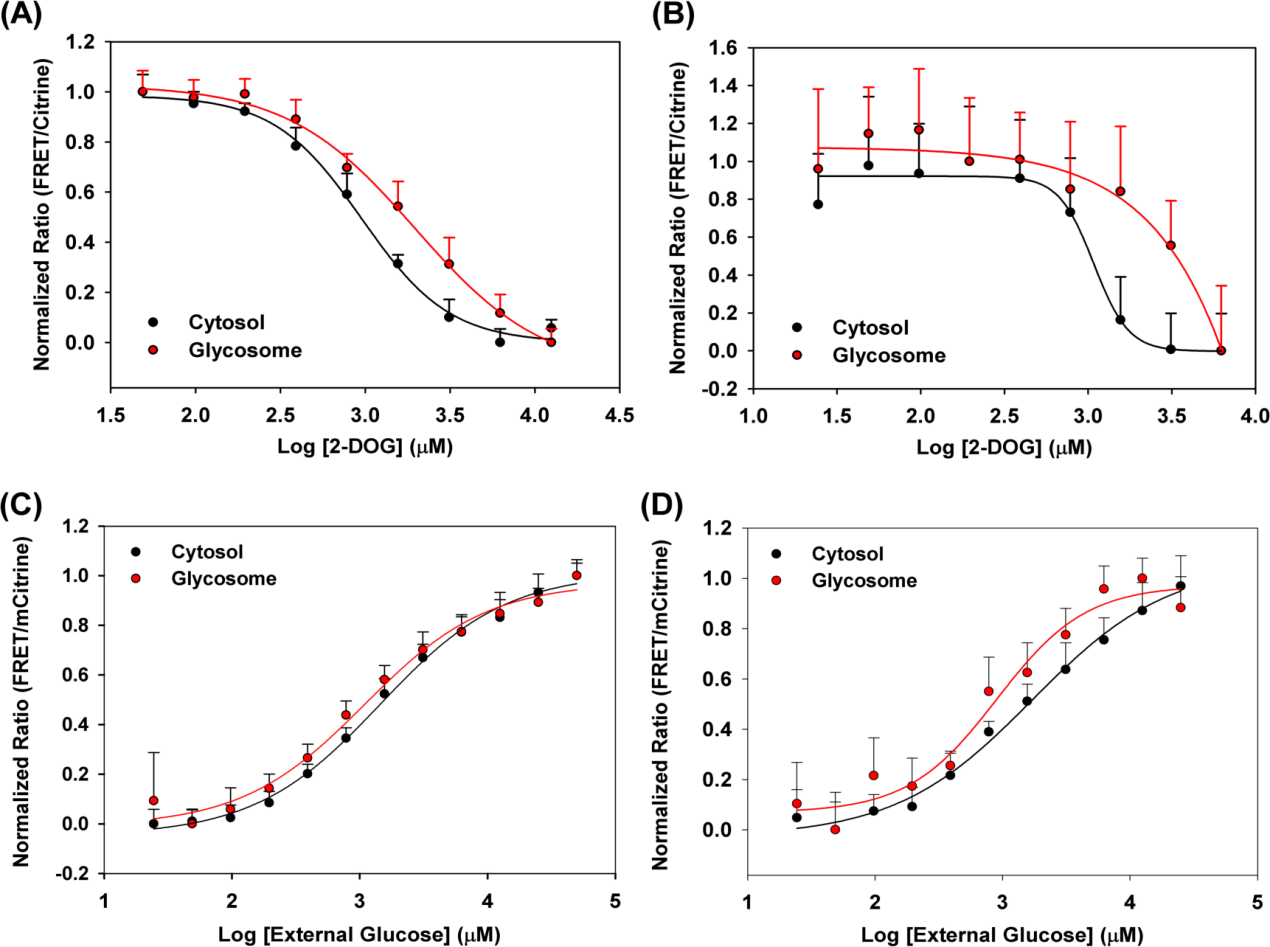
FLII^12^Pglu-700μδ6 and FLII^12^Pglu-700μδ6-PTS expressed *in vivo* responds to changes in internal glucose concentration. PCF (A) and BSF (B) parasites expressing FLII^12^Pglu-700μδ6 in cytosol (Black) or FLII^12^Pglu-700μδ6-PTS in glycosomes (Red) were incubated with 10mM glucose in PBS, 2-DOG concentration was titrated (.025–25,000 μM). Intracellular glucose was allowed to reach a steady state (45 minutes) before being analyzed via flow cytometry. FRET/mCitrine ratios resulting from glucose depletion were fitted using a single site binding isotherm to obtain apparent K_d_ values. Glucose dose response curves for PCF (C) and BSF (D) *T*. *brucei* expressing FLII^12^Pglu-700μδ6 (Cytosol, Black) or FLII^12^Pglu-700μδ6-PTS (Glycosomes, Red). Cells were incubated in varied extracellular glucose concentrations (0.05–50,000μM) and allowed to reach a steady state for 30 minutes before measurement of intracellular FRET ratios. FRET/mCitrine ratios were fitted to a single site binding isotherm and the K_app_ was calculated. Error bars represent symmetric standard deviation of n = 3 replicates.

To demonstrate that the FRET sensor reports changes in intracellular glucose concentration, BSF and PCF *T*. *brucei* were treated with 2-DOG, a small molecule that competes for uptake, thereby decreasing intracellular glucose concentrations. As shown in Fig 4, both cytosolic and glycosomal FRET/mCitrine ratios were diminished in the presence of 2-DOG in PCF (Fig 4A) and BSF (Fig 4B) parasites, consistent with the idea that reductions in FRET ratios reflect decreased glucose concentrations in the cell.

The glucose sensor signal of PCF and BSF parasites expressing FLII^12^Pglu-700μδ6 were monitored as a function of external glucose to score cytosolic and glycosomal glucose levels (Fig 4C & 4D). Increasing extracellular glucose increases the observed FRET ratio in a dose dependent manner, indicating that the glucose levels in the biosensor microenvironment also increase under these conditions. Apparent K_d_ (K_app_) for cytosol and glycosomes in PCF parasites were 1500 ± 150μM and 1100 ± 200μM, BSF parasites K_app_ were calculated as 1700 ± 300μM and 900 ± 200μM respectively. As we have shown, intracellular environment has little affect on the glucose response of the FLII12Pglu-700μδ6 biosensor. Therefore, K_app_ values can be used to make estimates about relative cytosolic and glycosomal glucose levels at external concentrations that are similar to the intracellular K_d_ of the biosensor. K_app_ values higher (or lower) than the calibrated biosensor K_d_ would indicate that different external glucose levels are required for intracellular glucose levels to reach glucose levels sufficient to reach the midpoint of biosensor response (i.e. 1000 μM). For example, the K_app_ for sensor response inside the glycosome of PCF and BSF parasites is different than the intracellular sensor response in permeabilized parasites (K_app_ = 1500 ± 150 μM and 1700 ± 300μM, respectively vs. 1000 ± 90 μM), an observation that suggests that glucose concentrations in the cytosol are lower than external values. Interestingly, cytosolic and glycosomal K_app_ are different in both life stages, indicating that glucose levels in the two compartments respond independently to changes in external glucose concentration.

**Fig 5:**
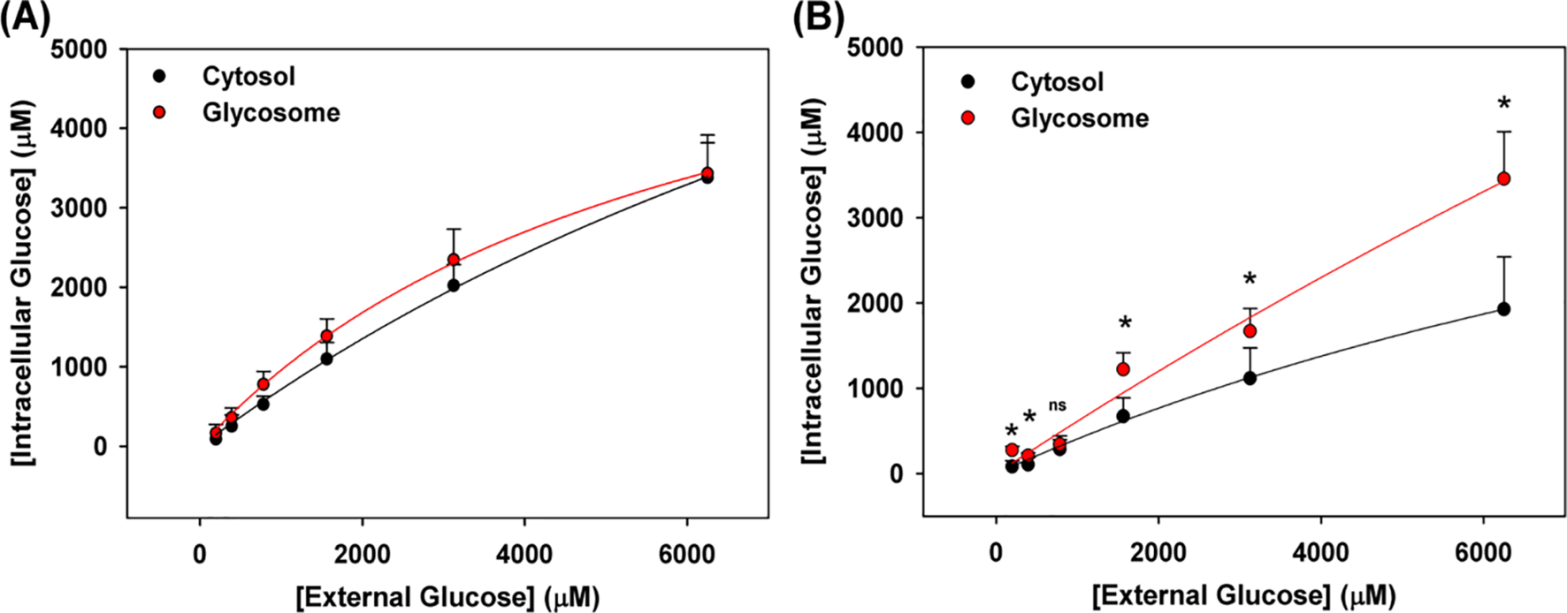
Calculated cytosolic and glycosomal glucose concentration in response to external glucose. PCF and BSF *T*. *brucei* expressing FLII^12^Pglu-700μδ6 in cytosol (Black) or FLII^12^Pglu-700μδ6-PTS in glycosomes (Red) were incubated in PBS and glucose was titrated (.025–25,000 μM). FRET signal in response to external glucose concentrations were transfomed into corresponding glucose concentrations and plotted versus environmental glucose. Resultant intracellular glucose concentrations for PCF (A) and BSF (B) were plotted versus the external glucose concentrations. Error bars represent symmetric standard deviation of n = 3 replicates. Cytosolic and glycosomal glucose concentrations were compared using One Way ANOVA, p < 0.05 were considered significant.

To quantitively measure intracellular glucose concentration, FRET responses from cytosolic and glycosomal sensors were calibrated to reflect corresponding intracellular glucose concentration(s). Biosensors with a single analyte binding site (including FLII^12^Pglu-700μδ6), have a two log linear response centered around the K_d_ of the sensor [26]. A full sensor dynamic range *in vivo* indicates that resultant FRET responses can be calibrated using the *in vivo* biosensor K_d_, together with the maximum and minimum biosensor response, using established sensor calibration protocols (Eq 1) [26]. Fig 4C and 4D show that the FLII^12^Pglu-700μδ6 responds over two logs of external glucose concentration, meaning that the full dynamic range of the sensor is observed over the external glucose concentrations probed. Using these methodologies, the FRET ratio in response to external glucose were converted into intracellular glucose concentrations (Fig 5).

### Cytosolic and glycosomal response to extracellular glucose

Fig 5A shows the resultant calibrated cytosolic and glycosomal glucose concentration in response to external glucose concentration in PCF trypanosomes. At external glucose concentrations below 1 mM, glycosomal and cytosolic glucose concentrations are not statistically different from each other, suggesting that at these conditions the cytosol and glycosome do not regulate glucose dependently. Above external glucose concentrations of 1mM, glycosomal glucose concentrations maintain concentrations that are slightly (1.2–1.3 fold) higher than the surrounding cytosol. The cytosol and glycosomes of PCF maintains glucose concentration roughly 50–70% of the external glucose, as external glucose increases the differences between intracellular and extracellular glucose increases. This suggests that as external glucose increases that glucose uptake into the cell becomes saturated and starts to approach a maximum rate. At concentrations, similar to physiological conditions in the mammalian host (6.25mM), cytosolic and glycosomal concentrations in PCF parasites are near saturated and maintain indistinguishable glucose concentrations of 3.4 ± 0.4 mM and 3.4 ± 0.5 mM respectively.

BSF parasites maintain intracellular glucose concentrations that are different than their PCF parasite counterparts, as shown in Fig 5B. As for the PCF data shown above, intracellular glucose concentrations in BSF parasites were quantified based on observed calibrated biosensor response. We then compared cytosolic and glycosomal glucose concentrations to external glucose concentrations. BSF parasites maintain cytosolic glucose concentration between 30–40% of the external glucose concentration. At 6.25mM external glucose, this correspondence equates to 1.9 ± 0.6 mM cytosolic glucose. Notably, this value is significantly lower than that observed in the cytosol of PCF parasites.

Under most extracellular glucose concentrations, PCF parasites and BSF parasites maintain higher glucose concentrations in the glycosome than in the cytosol; quantified concentrations range from 50–80% of the external environment, depending on the external glucose concentration. At external glucose concentrations of 6.25mM, the glucose concentration of the BSF intraglycosomal fluid is 3.5 ± 0.5 mM. Notably, over the range of external glucose concentrations tested, glycosomal glucose concentration was significantly higher in the glycosome versus the cytosol. At lower external glucose concentrations, glycosomal glucose is maintained 1-2-fold higher than the adjacent cytosolic levels. At physiological glucose conditions (6.25mM), glucose concentration in glycosomes maintain glucose concentrations that are ~1.8 times that of the cytosol. These data suggest that glucose transport is not freely diffusing across the glycosomal membrane and glucose in concentrated in the glycosome of BSF parasites.

Our measurements show that PCF and BSF maintain glucose concentrations differently at the same external glucose concentrations. At glucose concentrations, similar to physiological conditions (6.25mM), cytosolic glucose for PCF and BSF parasites were 3.4 ± 0.4 mM and 1.9 ± 0.6 mM respectively (Table 2). This observation indicates that cytosolic glucose concentrations in PCF parasites are maintained ~ 1.8-fold higher in the cytosol of PCF parasites versus BSF parasites at the same external glucose concentration. In contrast, glycosomal glucose levels are maintained at similar levels in PCF and BSF parasites, each roughly ~50% of the external glucose concentration, 3.4 ± 0.5 mM and 3.5 ± 0.5 mM respectively (Table 2).

**Table 2:** Intracellular glucose concentration in PCF and BSF parasites.

	Glucose Concentration^1^	% External Glucose^2^	Fold Increase^3^	
Cell Type				
Procyclic Cytosol	3.4 ± 0.4 mM	54%		
Procyclic Glycosome	3.4 ± 0.5 mM	54%	1	
Bloodstream Cytosol	1.9 ±0.6 mM	30%		
Bloodstream Glycosome	3.5 ± 0.5 mM	56%	1.8	

^1^Intracellular glucose concentration at an external glucose concentration of 6.25 mM.

^2^Compared to 6.25mM external glucose.

^3^Fold increase in glycosomal glucose versus cytosolic concentration.

## Discussion

By measuring intracellular glucose with FRET-based sensors we have, for the first time, characterized the relationship between environmental, cytosolic, and glycosomal glucose concentrations in living trypanosomes. Our measurements show that PCF and BSF parasites maintain cytosolic and glycosomal glucose concentrations differently. Interestingly, our observations show that PCF *T*. *brucei* maintains cytosolic glucose levels ~1.8 times higher than those measured in the cytosol of BSF parasites. The observed differences between PCF and BSF cytosolic glucose distribution likely reflects the differences in how the two life stages transport and process glucose.

The *T*. *brucei* genome encodes two hexose transporters, THT1 and THT2, both of which are members of the facilitated glucose transporter GLUT1 family [34] and are known to localize to the cell membrane. BSF parasites primarily express THT1 in the cell membrane, which has lower affinity for substrate than THT2, making it suitable for glucose acquisition in the glucose rich environment of the mammalian bloodstream. PCF parasites predominantly express the higher affinity THT2 [35],[34], a transport system optimized to acquire the hexose from the relatively glucose-depleted environs of the tsetse fly [35]. THT isoform expression differences could explain the lower cytosolic glucose concentrations in BSF parasites versus PCF cells at equivalent extracellular glucose concentrations.

In BSF parasites, glycosomal glucose is ~1.8-fold concentrated in the glycosome compared to the surrounding cytosol. Although mechanisms for this concentrating process have not been described in previous literature, one possible explanation is that glucose transport into the glycosome is an active process that results in the observed concentration gradient. Enthusiasm for this possibility should be tempered, however, given the lack of obvious candidate transporters. In peroxisomes, pores that selectively allow inorganic ions and hydrophilic metabolites to pass while blocking other molecules including ATP have been described [36]. Glycosomes also harbor pores with behavior that suggests that they are water-filled in the membrane and potentially "non-selective" channels [11], but the role of such channels in glucose homeostasis has not been resolved.

We are uniquely equipped with methods capable of identifying the cellular machinery behind the uptake and distribution of glucose. Using this tool in in concert with forward and reverse molecular genetics and chemical inhibitors, the identity of components of glucose subcellular distribution will be resolved. Proteins involved in glucose distribution into the glycosome would be interesting targets for anti-trypanosome therapies, given the parasites reliance on glucose metabolism in the glycosome [2][37].

## Supporting information

S1 Fig FLII^12^Pglu-700μδ6-PTS colocalizes with aldolase in glycosomes. Immunofluorescence (IF) microscopy of BSF and PCF *T*. *brucei* parasites harboring FLII^12^Pglu-700μδ6-PTS. IF microscopy was performed using α Aldolase and α GFP (M3E6) antisera. DAPI was added to stain the nucleus and kinetoplast DNA. Scale bar = 5 μm.
(TIF)

S2 Fig Glycosome bound protease protection assay. Protease protection assay of procyclic form parasites constitutively expressing the FLII12Pglu-700μδ6-PTS glucose sensor. 10 6 cells were permeabilized with digitonin and incubated with water (lane 1), proteinase K (lane 2), Triton X-100 (lane 3) or both proteinase K and Triton X-100 (lane 4) for 30 minutes on ice. Following incubation, proteins were precipitated with trichloroacetic acid and separated by SDS-PAGE. Protein was detected by western blot using anti-GFP antibodies.
(TIF)

S3 Fig *In vitro* glucose response curve of purified FLII^12^Pglu-700μδ6-PTS. *In vitro* calibration curve of bacterially expressed and purified FLII^12^Pglu-700μδ6-PTS in PBS titrated with glucose 1–50,000 μM glucose. FRET ratio was collected via fluorometer (430nm excitation 533nm emission for FRET, 433nm excitation 480 nm emission for ECFP) for each glucose concentration. An increase in FRET ratio indicates an increase in glucose concentration. Data were fitted to a single site binding isotherm, calculated K_d_ for glucose was calculated as 880 ± 50 μM; error bars represent standard deviation from n = 3 samples.
(TIF)

S4 Fig Glucose-6-phospate does not affect FLII12Pglu-700μδ6-PTS. Purified FLII12Pglu-700μδ6-PTS incubated with varying glucose (black bars) and glucose-6-phosphate (red) in PBS. FLII12Pglu-700μδ6 FRET ratio was collected via fluorometer (430nm excitation 533nm emission for FRET, 433nm excitation 480 nm emission for ECFP) for each glucose concentration. An increase in FRET ratio indicates an increase in sensor binding. Error bars represent standard deviation for n = 3 replicates.
(TIF)

S5 Fig Gating scheme used for flow cytometry analysis. Cells used for analysis were gated for three criteria. Bivariate plot of FSC:H versus FSC:A (A) was used to exclude double and dividing cells. Cells whose FSC:H and FSC:A are not approximately equal are double and/or dividing cells, singlet cell gates are gated accordingly. Dead cells were then removed using a SSC:A versus FSC:A bivariate plot (B). Dead cells and debris scatter less light in FSC:A than living healthy cells; these characteristics were used to place the live cell gate. Once doublets and dead cells were excluded, cells were then gated off of their fluorescence intensities in the FRET and mCitrine channels (C).
(TIF)

S6 Fig β-escin selectively permeabilizes cell membranes to small molecules. Bivariate plots of WT PCF cells (A) and PCF cells expressing FLII^12^Pglu-700μδ6 (B). Cells partially permeabilized with β-escin (C) are represented by the double positive population for mCitrine and PI, representing cells that allow small molecules (i.e. PI or glucose) into the cell, but do not allow FLII^12^Pglu-700μδ6 (and other larger molecules) out of the cell. These cells were used for *in vivo* calibration of endogenously expressed FLII^12^Pglu-700μδ6. Cells permeabilized with high concentrations of β-escin (D), were devoid of all protein sensor due to more complete permeabilization.
(TIF)

S7 Fig TbHK1 activity is not impacted by the presence of FLII^12^Pglu-700μδ6. Recombinant TbHK1 activity was monitored in vitro in the presence of increasing amounts of FLII12Pglu-700μδ6 in order to achieve TbHK1 to sensor molar ratios of 1 to 0.5, 1, 2, 4, 8, and 10, respectively. K2354, a known benzamidobenzoic acid inhibitor of TbHK1 [31], was used at 10 μM as a control.
(TIF)

## References

[1] Kennedy PGE. The continuing problem of human African trypanosomiasis (sleeping sickness). Ann Neurol. 2008;64: 116–26. doi: 10.1002/ana.21429 18756506 10.1002/ana.21429

[2] Fairlamb AH. Chemotherapy of human African trypanosomiasis: Current and future prospects. Trends Parasitol. 2003;19: 488–494. doi: 10.1016/j.pt.2003.09.002 14580959 10.1016/j.pt.2003.09.002

[3] Brun R, Schumacher R, Schmid C, Kunz C, Burri C. The phenomenon of treatment failures in human African trypanosomosis. Trop Med Int Heal. 2001;6: 906–914. doi: 10.1046/j.1365-3156.2001.00775.x 10.1046/j.1365-3156.2001.00775.x 11703845

[4] Robays J, Nyamowala G, Sese C, Kande VBKM, Lutumba P, Van Der Veken W, et al High failure rates of melarsoprol for sleeping sickness, Democratic Republic of Congo. Emerg Infect Dis. 2008;14: 966–967. doi: 10.3201/eid1406.071266 18507916 10.3201/eid1406.71266 PMC2600279

[5] Visser N, Opperdoes FR. Glycolysis in Trypanosoma brucei. Eur J Biochem. 1980;103: 623–32. Available: http://www.ncbi.nlm.nih.gov/pubmed/6766864 6766864 10.1111/j.1432-1033.1980.tb05988.x

[6] Van Grinsven KW a, Van Den Abbeele J, Van Den Bossche P, Van Hellemond JJ, Tielens AGM. Adaptations in the glucose metabolism of procyclic Trypanosoma brucei isolates from tsetse flies and during differentiation of bloodstream forms. Eukaryot Cell. 2009;8: 1307–1311. doi: 10.1128/EC.00091-09 19542311 10.1128/EC.00091-09 PMC2725551

[7] Kuile BH. Adaptation of metabolic enzyme activities of Trypanosoma brucei promastigotes to growth rate and carbon regimen. Adaptation of Metabolic Enzyme Activities of Trypanosoma brucei Promastigotes to Growth Rate and Carbon Regimen. 1997;179: 4699–4705. 10.1128/jb.179.15.4699-4705.1997 PMC179314 9244255

[8] Michels P a M, Bringaud F, Herman M, Hannaert V. Metabolic functions of glycosomes in trypanosomatids. Biochim Biophys Acta. 2006;1763: 1463–77. doi: 10.1016/j.bbamcr.2006.08.019 17023066 10.1016/j.bbamcr.2006.08.019

[9] Antonenkov VD, Grunau S, Ohlmeier S, Hiltunen JK. Peroxisomes are oxidative organelles. Antioxid Redox Signal. 2010;13: 525–537. doi: 10.1089/ars.2009.2996 19958170 10.1089/ars.2009.2996

[10] Colasante C, Voncken F, Manful T, Ruppert T, Tielens AGM, van Hellemond JJ, et al Proteins and lipids of glycosomal membranes from Leishmania tarentolae and Trypanosoma brucei. F1000Research. 2013;2: 27 doi: 10.12688/f1000research.2-27.v1 24358884 10.12688/f1000research.2-27.v1 PMC3814921

[11] Gualdron-López M, Vapola MH, Miinalainen IJ, Hiltunen JK, Michels PAM, Antonenkov VD. Channel-forming activities in the glycosomal fraction from the bloodstream form of trypanosoma brucei. PLoS One. 2012;7 doi: 10.1371/journal.pone.0034530 22506025 10.1371/journal.pone.0034530 PMC3323538

[12] Shaner NC, Steinbach PA, Tsien RY. A guide to choosing fluorescent proteins. Nat Methods. 2005;2: 905–909. doi: 10.1038/nmeth819 16299475 10.1038/nmeth819

[13] Liemburg-Apers DC, Imamura H, Forkink M, Nooteboom M, Swarts HG, Brock R, et al Quantitative glucose and ATP sensing in mammalian cells. Pharm Res. 2011;28: 2745–57. doi: 10.1007/s11095-011-0492-8 21691894 10.1007/s11095-011-0492-8

[14] Persechini A, Lynch JA, Romoser VA. Novel fluorescent indicator proteins for monitoring free intracellular Ca2+. Cell Calcium. 1997;22: 209–216. 9330791 10.1016/s0143-4160(97)90014-2

[15] Hitomi Takanaga, Bhavna Chaudhuri WBF. GLUT1 and GLUT9 as the major contributors to glucose influx in HEPG2 cells identified by a high sensitivity intramolecular FRET glucose sensor. Science (80-). 2009;1778: 1091–1099. doi: 10.1016/j.bbamem.2007.11.015.GLUT1 10.1016/j.bbamem.2007.11.015 PMC2315637 18177733

[16] Journal T, Dec N. Continuous Cultivation of Trypanosoma brucei Blood Stream Forms in a Medium Containing a Low Concentration of Serum Protein without Feeder Cell Layers Hiroyuki Hirumi; Kazuko Hirumi Continuous Cultivation of Trypanosomabrucei Blood Stream Forms in a Medi. J Parasitol. 1989;75: 985–989. 2614608

[17] Zeitschrift A, Band AT, Link P, Dienst E, Eth E. Cultivation of vertebrate infective forms derived from metacyclic forms of pleomorphic “Trypanosoma brucei” stocks: short communication. Acta Trop. 1979; 387–390. 44103

[18] Fehr M, Frommer WB, Lalonde S. Visualization of maltose uptake in living yeast cells by fluorescent nanosensors. Proc Natl Acad Sci U S A. 2002;99: 9846–51. doi: 10.1073/pnas.142089199 12097642 10.1073/pnas.142089199 PMC125039

[19] Wang Z, Morris JC, Drew ME, Englund PT, Acad EPN, Sci USA. Inhibition of Trypanosoma brucei Gene Expression by RNA Interference Using an Integratable Vector with Opposing T7 Promoters * RNA interference is a powerful method for inhibition. J Biol Chem. 2000;275: 40174–40179. doi: 10.1074/jbc.M008405200 11013266 10.1074/jbc.M008405200

[20] Burkard G, Fragoso CM, Roditi I. Highly efficient stable transformation of bloodstream forms of Trypanosoma brucei. Mol Biochem Parasitol. 2007;153: 220–223. doi: 10.1016/j.molbiopara.2007.02.008 17408766 10.1016/j.molbiopara.2007.02.008

[21] Roggy JL, Bangs JD. Molecular cloning and biochemical characterization of a VCP homolog in African trypanosomes. 1999;98: 1–15. 10029305 10.1016/s0166-6851(98)00114-5

[22] Alexander DL, Schwartz KJ, Balber AE, Bangs JD. Developmentally regulated trafficking of the lysosomal membrane protein p67 in Trypanosoma brucei. J Cell Sci. 2002; 3253–3263. 12140257 10.1242/jcs.115.16.3253

[23] Gould SJ, Keller G a, Hosken N, Wilkinson J, Subramani S. A conserved tripeptide sorts proteins to peroxisomes. J Cell Biol. 1989;108: 1657–64. Available: http://www.pubmedcentral.nih.gov/articlerender.fcgi?artid=2115556&tool=pmcentrez&rendertype=abstract 2654139 10.1083/jcb.108.5.1657 PMC2115556

[24] Opperdoes FR, Borst P. LOCALIZATION OF NINE GLYCOLYTIC ENZYMES IN A MICROBODY-LIKE ORGANELLE IN TRYPANOSOMA BRUCEI: THE GLYCOSOME. FEBS Lett. 1977;80: 360–364. 142663 10.1016/0014-5793(77)80476-6

[25] McNew JA, Goodman JM. The targeting and assembly of peroxisomal proteins: Some old rules do not apply. Trends Biochem Sci. 1996;21: 54–58. doi: 10.1016/0968-0004(96)80866-8 8851661

[26] Cazzulo J. Aerobic fermentation of glucose by trypanosomatids. FASEB. 1992;6: 3153–3161. 10.1096/fasebj.6.13.1397837 1397837

[27] Giordani M. Surgical solution of intractable ascites in 3 series of patients treated by continuous peritoneovenous shunting. Minerva Chir. 1989;44: 457–461. doi: 10.1016/j.ymeth.2008.09.015.Measuring 2654717

[28] McCombs JE, Palmer AE. Measuring calcium dynamics in living cells with genetically encodable calcium indicators. Methods. Elsevier Inc.; 2008;46: 152–159. doi: 10.1016/j.ymeth.2008.09.015 18848629 10.1016/j.ymeth.2008.09.015 PMC2654717

[29] Kovacic PB, Chowdhury HH, Velebit J, Kreft M, Zorec R. New Insights into Cytosolic Glucose Levels during Differentiation of 3T3-L1 Fibroblasts into Adipocytes *. 2011;286: 13370–13381. doi: 10.1074/jbc.M110.200980 21349852 10.1074/jbc.M110.200980 PMC3075683

[30] Morris MT, DeBruin C, Yang Z, Chambers JW, Smith KS, Morris JC. Activity of a second Trypanosoma brucei hexokinase is controlled by an 18-amino-acid C-terminal tail. Eukaryot Cell. 2006;5: 2014–2023. doi: 10.1128/EC.00146-06 17028241 10.1128/EC.00146-06 PMC1694814

[31] Flaherty DP, Harris MT, Schroeder CE, Khan H, Kahney EW, Hackler AL, et al Optimization and Evaluation of Antiparasitic Benzamidobenzoic Acids as Inhibitors of Kinetoplastid Hexokinase1. ChemMedChem. 2017;27599: 1994–2005. doi: 10.1002/cmdc.201700592 10.1002/cmdc.201700592 PMC5808564 29105342

[32] Lin S, Morris MT, Ackroyd PC, Morris JC, Christensen K a. Peptide-targeted delivery of a pH sensor for quantitative measurements of intraglycosomal pH in live Trypanosoma brucei. Biochemistry. 2013;52: 3629–37. doi: 10.1021/bi400029m 23651061 10.1021/bi400029m PMC5507074

[33] Griesbeck O, Baird GS, Campbell RE, Zacharias DA, Tsien RY. Reducing the Environmental Sensitivity of Yellow Fluorescent. 2001;276: 29188–29194. doi: 10.1074/jbc.M102815200 11387331 10.1074/jbc.M102815200

[34] Saignat L, Cedex B. Differential Regulation of Two Distinct Families of Glucose Transporter Genes in Trypanosoma brucei. Mol Cell Biol. 1993;13: 1146–1154. 8423781 10.1128/mcb.13.2.1146-1154.1993 PMC358999

[35] Barrett MP, Tetaud E, Seyfang A, Bringaud F, Baltz T. Trypanosome glucose transporters. Mol Biochem Parasitol. 1998;91: 195–205. doi: 10.1016/S0166-6851(97)00192-8 9574935 10.1016/s0166-6851(97)00192-8

[36] Antonenkov VD. The rat liver peroxisomal membrane forms a permeability barrier for cofactors but not for small metabolites in vitro. J Cell Sci. 2004;117: 5633–5642. doi: 10.1242/jcs.01485 15509867 10.1242/jcs.01485

[37] Parsons M. Glycosomes: parasites and the divergence of peroxisomal purpose. Mol Microbiol. 2004;53: 717–24. doi: 10.1111/j.1365-2958.2004.04203.x 15255886 10.1111/j.1365-2958.2004.04203.x

