# Supplementary figures and images for: A FRET flow cytometry method for monitoring cytosolic and glycosomal glucose in living kinetoplastid parasites
Source: PLoS Negl Trop Dis. 2018 May 31;12(5):e0006523. doi: 10.1371/journal.pntd.0006523 (PMC5997345; doi:10.1371/journal.pntd.0006523)

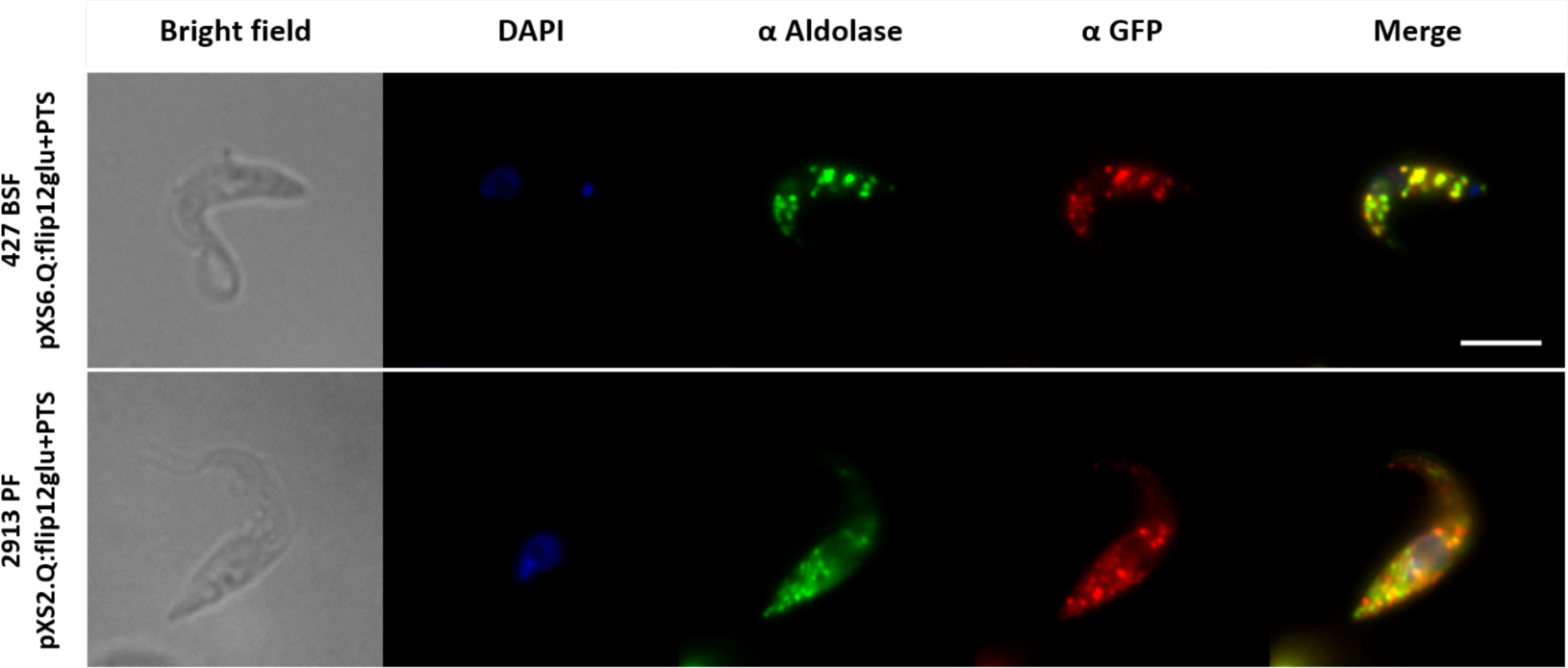

Supplement: S1 Fig — Immunofluorescence (IF) microscopy of BSF and PCF T. brucei parasites harboring FLII12Pglu-700μδ6-PTS. IF microscopy was performed using α Aldolase and α GFP (M3E6) antisera. DAPI was added to stain the nucleus and kinetoplast DNA. Scale bar = 5 μm. (TIF) [file pntd.0006523.s001.tif]

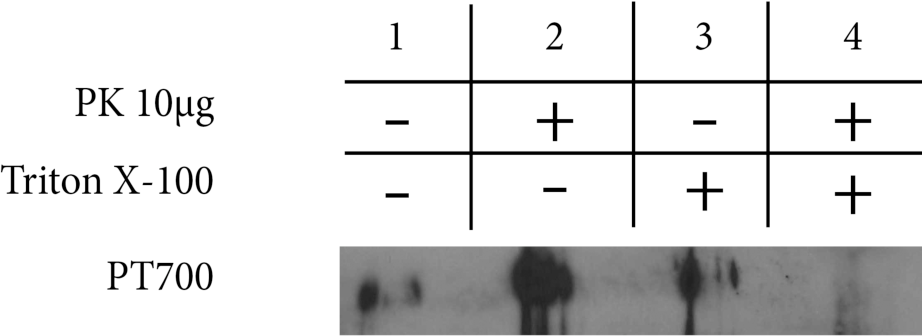

Supplement: S2 Fig — Protease protection assay of procyclic form parasites constitutively expressing the FLII12Pglu-700μδ6-PTS glucose sensor. 10 6 cells were permeabilized with digitonin and incubated with water (lane 1), proteinase K (lane 2), Triton X-100 (lane 3) or both proteinase K and Triton X-100 (lane 4) for 30 minutes on ice. Following incubation, proteins were precipitated with trichloroacetic acid and separated by SDS-PAGE. Protein was detected by western blot using anti-GFP antibodies. (TIF) [file pntd.0006523.s002.tif]

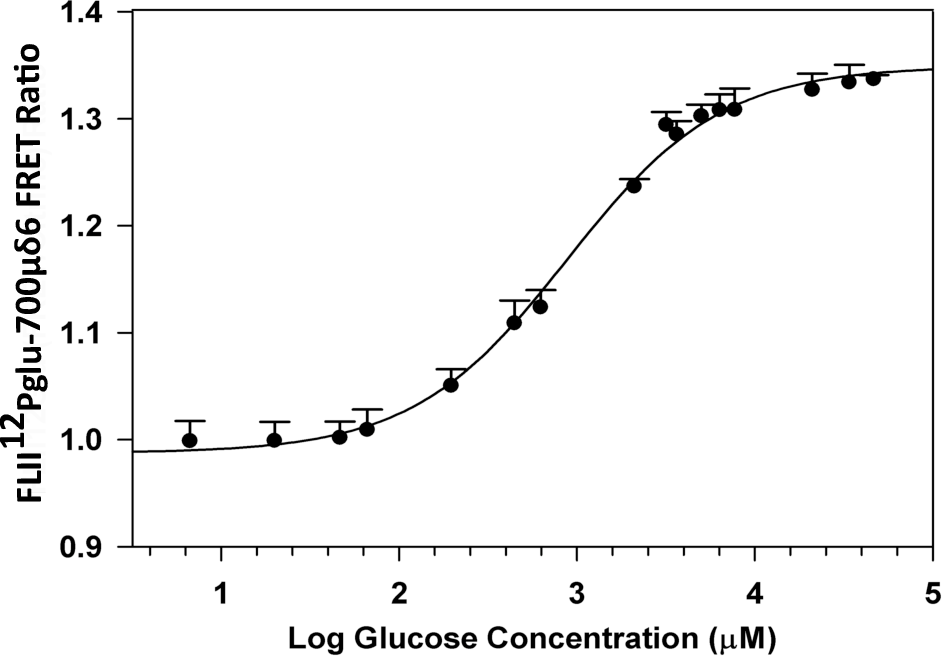

Supplement: S3 Fig — In vitro calibration curve of bacterially expressed and purified FLII12Pglu-700μδ6-PTS in PBS titrated with glucose 1–50,000 μM glucose. FRET ratio was collected via fluorometer (430nm excitation 533nm emission for FRET, 433nm excitation 480 nm emission for ECFP) for each glucose concentration. An increase in FRET ratio indicates an increase in glucose concentration. Data were fitted to a single site binding isotherm, calculated Kd for glucose was calculated as 880 ± 50 μM; error bars represent standard deviation from n = 3 samples. (TIF) [file pntd.0006523.s003.tif]

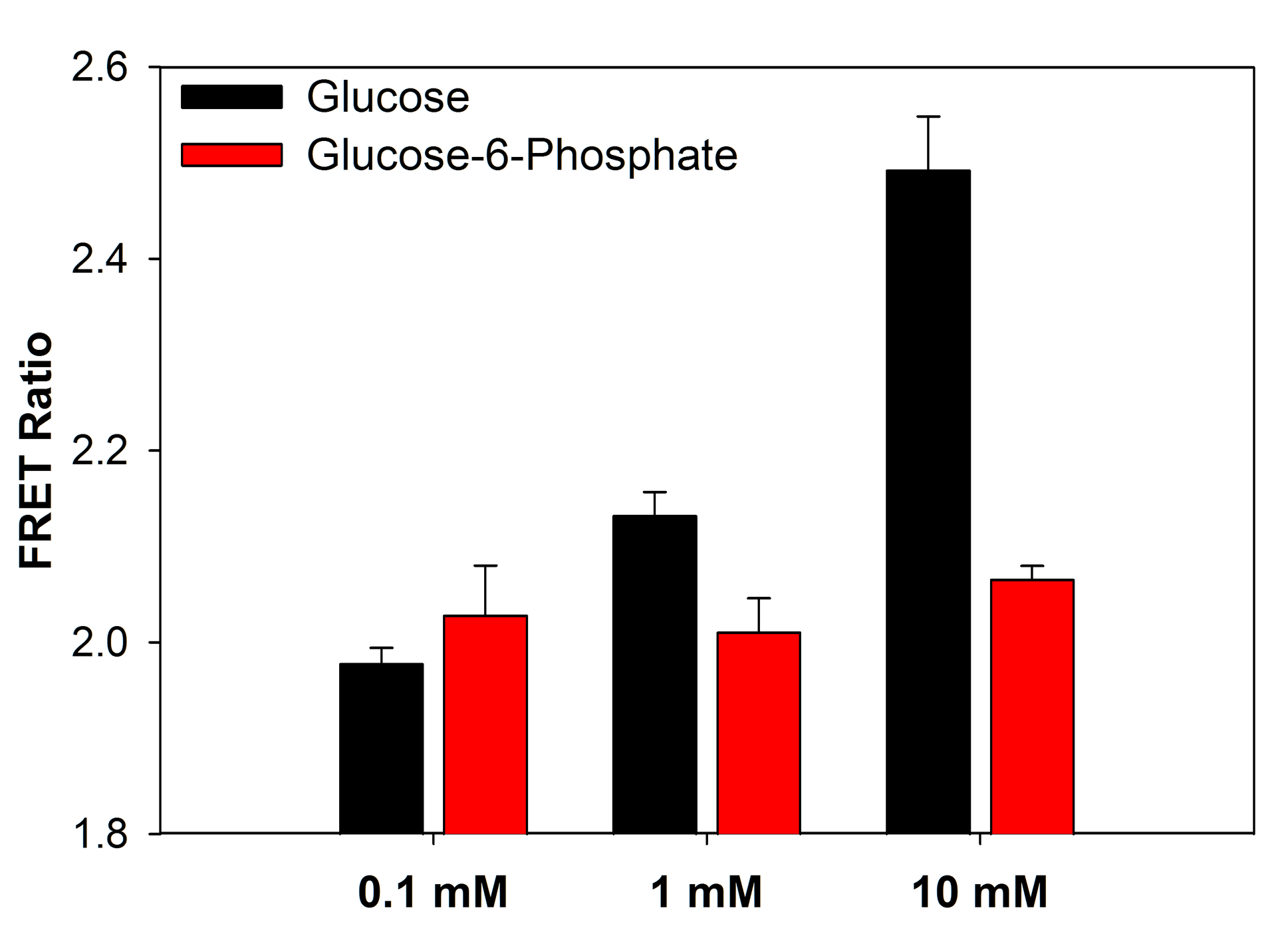

Supplement: S4 Fig — Purified FLII12Pglu-700μδ6-PTS incubated with varying glucose (black bars) and glucose-6-phosphate (red) in PBS. FLII12Pglu-700μδ6 FRET ratio was collected via fluorometer (430nm excitation 533nm emission for FRET, 433nm excitation 480 nm emission for ECFP) for each glucose concentration. An increase in FRET ratio indicates an increase in sensor binding. Error bars represent standard deviation for n = 3 replicates. (TIF) [file pntd.0006523.s004.tif]

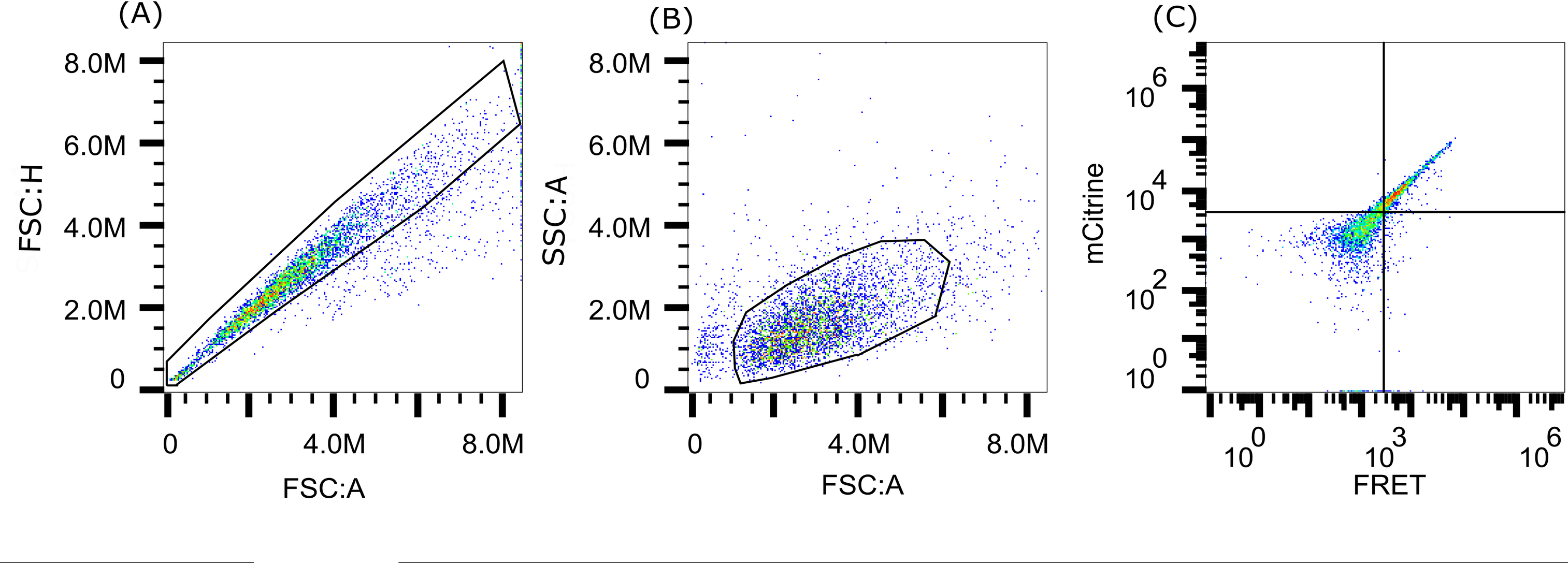

Supplement: S5 Fig — Cells used for analysis were gated for three criteria. Bivariate plot of FSC:H versus FSC:A (A) was used to exclude double and dividing cells. Cells whose FSC:H and FSC:A are not approximately equal are double and/or dividing cells, singlet cell gates are gated accordingly. Dead cells were then removed using a SSC:A versus FSC:A bivariate plot (B). Dead cells and debris scatter less light in FSC:A than living healthy cells; these characteristics were used to place the live cell gate. Once doublets and dead cells were excluded, cells were then gated off of their fluorescence intensities in the FRET and mCitrine channels (C). (TIF) [file pntd.0006523.s005.tif]

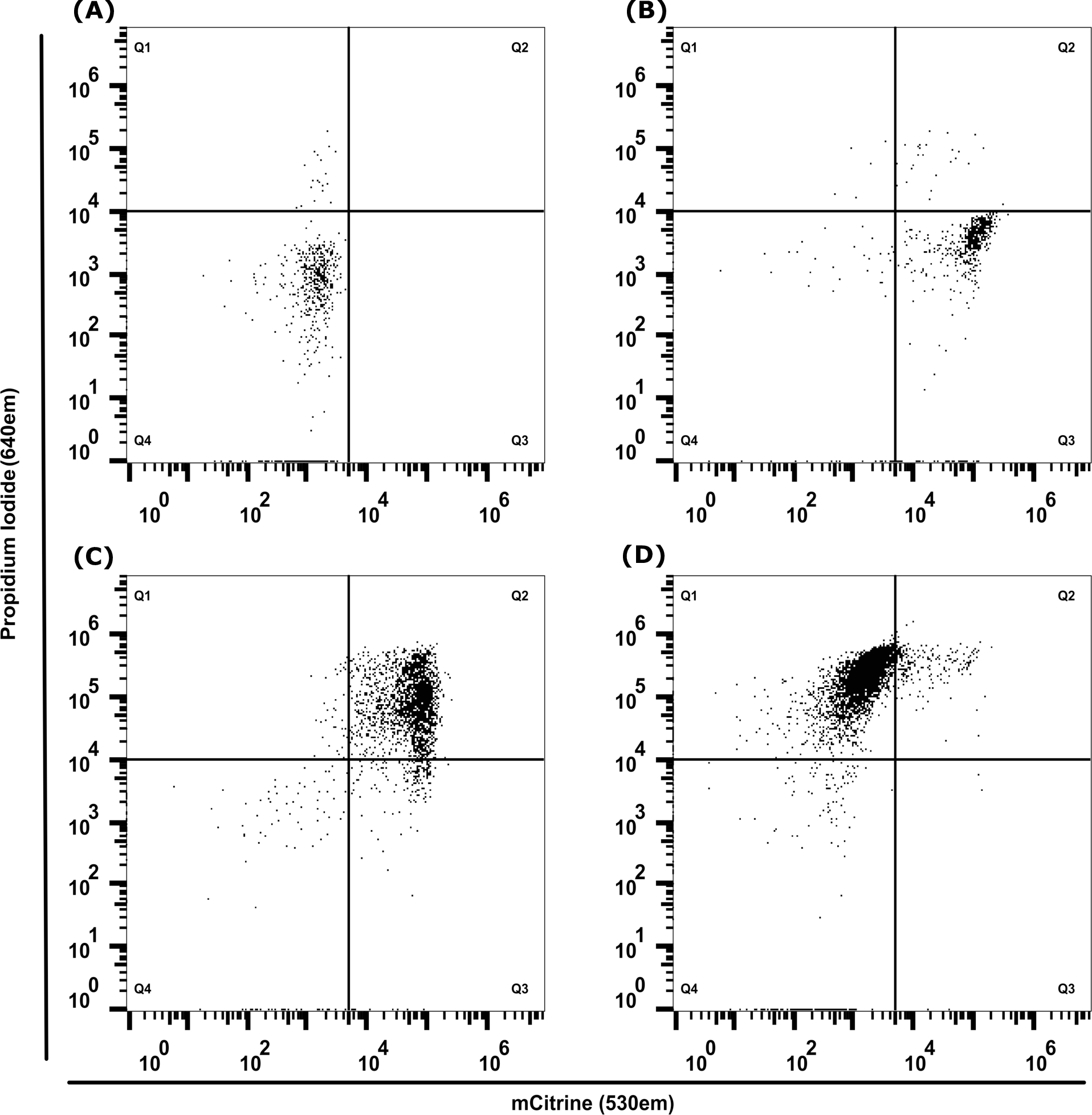

Supplement: S6 Fig — Bivariate plots of WT PCF cells (A) and PCF cells expressing FLII12Pglu-700μδ6 (B). Cells partially permeabilized with β-escin (C) are represented by the double positive population for mCitrine and PI, representing cells that allow small molecules (i.e. PI or glucose) into the cell, but do not allow FLII12Pglu-700μδ6 (and other larger molecules) out of the cell. These cells were used for in vivo calibration of endogenously expressed FLII12Pglu-700μδ6. Cells permeabilized with high concentrations of β-escin (D), were devoid of all protein sensor due to more complete permeabilization. (TIF) [file pntd.0006523.s006.tif]

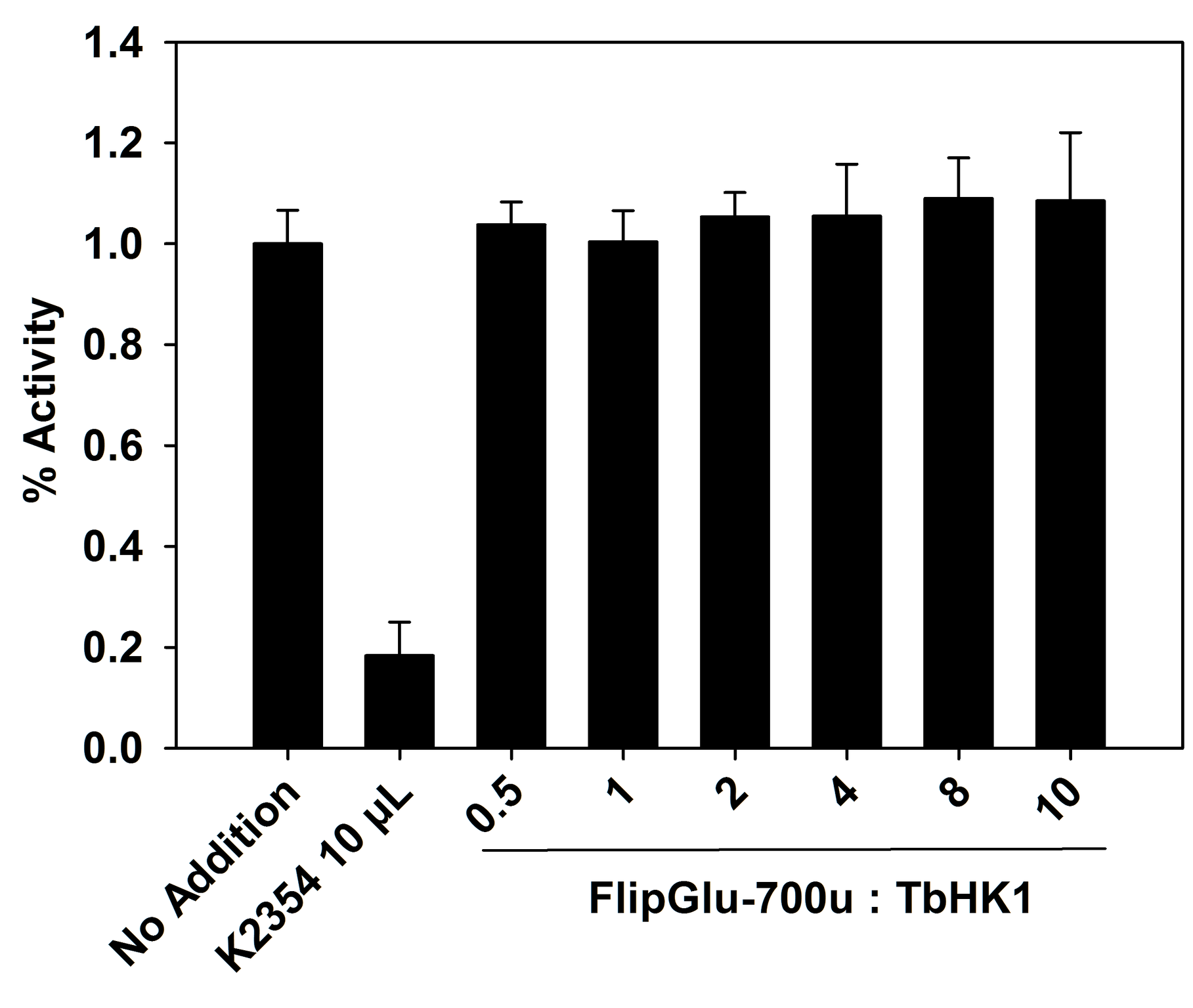

Supplement: S7 Fig — Recombinant TbHK1 activity was monitored in vitro in the presence of increasing amounts of FLII12Pglu-700μδ6 in order to achieve TbHK1 to sensor molar ratios of 1 to 0.5, 1, 2, 4, 8, and 10, respectively. K2354, a known benzamidobenzoic acid inhibitor of TbHK1 [31], was used at 10 μM as a control. (TIF) [file pntd.0006523.s007.tif]
